# A novel proliferation synergy factor cocktail maintains proliferation and improves transfection efficiency in muscle cells and fibroblasts under low-serum conditions

**DOI:** 10.3389/fcell.2025.1680263

**Published:** 2025-11-17

**Authors:** Jianan Li, Jingqi Huang, Zeyang Gao, Chunpeng He, Zaiyan Xu, Bo Zuo

**Affiliations:** 1 Key Laboratory of Swine Genetics and Breeding of the Ministry of Agriculture and Rural Affairs, Huazhong Agricultural University, Wuhan, China; 2 Key Laboratory of Agriculture Animal Genetics, Breeding and Reproduction of the Ministry of Education, Huazhong Agricultural University, Wuhan, China; 3 Department of Basic Veterinary Medicine, College of Veterinary Medicine, Huazhong Agricultural University, Wuhan, China; 4 Hubei Hongshan Laboratory, Wuhan, China

**Keywords:** low-serum culture, proliferation synergy factor cocktail, porcine skeletal muscle satellite cells, porcine kidney fibroblasts, cell proliferation, transfection efficiency

## Abstract

Low-serum culture systems offer enhanced controllability, improved safety, and increased cost-effectiveness for applications in tissue engineering, regenerative medicine, drug screening, and cultured meat production. In this study, we developed a novel proliferation synergy factor cocktail (PSFC) consisting of IGF-1, bFGF, TGF-β, IL-6, and G-CSF under low-serum (5% FBS) conditions. This system not only sustained robust proliferation of porcine muscle satellite cells (PSCs) and porcine kidney fibroblasts (PKFs), but also exhibited broad applicability in C2C12 myoblasts and mouse skeletal muscle satellite cells (SSCs). RT-qPCR and Western blot showed that there were no significant differences in the expression levels of the proliferation marker Ki67, as well as the myogenic regulatory factors MyoG and MyHC, between the 5% FBS-PSFC culture system and the conventional serum culture system. Notably, PSFC supplementation enhanced the average transfection efficiency by 16.9% across all tested cell types. Furthermore, the 5% FBS-PSFC platform facilitated three-dimensional (3D) culture within gelatin methacryloyl (GelMA) hydrogels, enabling scalable cultured meat production while reducing serum costs by 75%. Further RNA-seq analysis revealed that the there was no significant changes in the expression of cell proliferation-related genes which may be crucial for maintaining cell proliferation of this system, while the upregulation of genes associated with membrane fluidity and endocytosis, such as *ITGA3*, *SEMA7A*, *ADAM8* and *AREG*, may lead to the enhancement of transfection efficiency. Collectively, these findings establish a cost-effective and versatile culture platform that addresses critical challenges in cell expansion for cellular agriculture, while providing a scalable approach to enhance transfection efficiency for gene editing applications.

## 1 Introduction

Conventional cell culture systems primarily rely on high concentrations of 10%–20% fetal bovine serum (FBS) to provide essential nutrients for maintaining cell growth (Kolkmann et al., 2022). However, this reliance on serum presents evident limitations due to the unclear biological components of FBS and the associated risks of microbial or viral contamination (Pfeifer et al., 2024). Variations between serum batches can lead to an accelerated loss of stemness in key cell types, such as muscle stem cells, during prolonged culture (Zhu et al., 2022). In particular, adult stem cells that are capable of self-renewal and differentiation into multinucleated myotubes have been shown to gradually lose their stem cell characteristics in high serum environments as passage numbers increase (Ren et al., 2024). These comprehensive challenges underscore the urgent need for standardized, low-serum, or even serum-free culture platforms that can sustain cell functionality during large-scale expansion.

Recent advances in serum-free or low-serum media have demonstrated feasibility across cell types. For instance, a serum-free differentiation medium (SFDM) incorporating sodium bicarbonate, L-ascorbic acid (2-phosphate), epidermal growth factor-1 (EGF1), bovine serum albumin and a transferrin-insulin-LPA complex system have been shown to support the myogenic differentiation of bovine satellite cells without serum starvation (Messmer et al., 2022). Similarly, researchers enhanced the B8 basal medium by adding recombinant albumin along with L-ascorbic acid, insulin, transferrin, selenium (ITS), fibroblast growth factor-2 (FGF-2), neuregulin-1 (NRG-1), and transforming growth factor-β3 (TGF-β3). The resulting Beefy-9 medium supported long-term bovine satellite cell proliferation while maintaining myogenic potential (Stout et al., 2022). Although a wide variety of serum-free medium types are available on the market, deficiencies still exist. For instance, seven commercially available serum-free media and three supplements were tested to maintain the proliferation of bovine myoblasts, but the cell count did not reach the high levels observed in 10% or 20% FBS culture (Kolkmann et al., 2020). Similarly, a serum-free medium supplemented with *C. vulgaris* extract, two growth factors, and insulin was used to culture bovine fibroblasts. However, the cell growth and viability levels remained lower than those of the control group cultured with 10% FBS (Defendi-Cho and Gould, 2023). In addition, under most serum-free culture conditions, primary human mesenchymal stem cells (hMSCs) typically cannot adhere to conventional cell culture plates compared to the 10% FBS culture conditions (Kai et al., 2024). Moreover, the pig and bovine myoblasts, which maintained a poor cell state, were cultured in serum-free medium (B8), and the passage times of bovine myoblasts did not exceed two generations (Schenzle et al., 2025). Additionally, a serum-free medium containing 11 cytokines was designed using the Placket-Burman (PB) and Box-Behnken (BB) methods. Although this medium can effectively promote the proliferation of fish myoblasts, the large number of cytokines increases the production cost (Zhang et al., 2024).

Notably, the pursuit of low-serum formulations itself represents a critical and practical pathway in cell culture. Under current technological conditions, maintaining the normal state and proliferation of cells without FBS may be challenging. Therefore, the current optimal approach is to reduce the dosage of FBS, particularly to 5% FBS, which has been shown to effectively maintain the normal state and proliferation of cells. For example, under 5% and 10% FBS conditions, there was no significant difference in the proliferation ability of Larimichthys crocea Muscle Satellite Cells (LCMSCs), indicating that these cells have adapted to the 5% serum condition (Wei et al., 2025). Additionally, low serum medium (5% FBS) can be used for the expansion of Madin Darby Canine Kidney Cells (MDCK) and the proliferation of the influenza virus. This method has the potential to reduce costs while ensuring high production and quality of vaccines (Cai et al., 2024). However, the concentrations of FBS lower than 5% performed poorly in maintaining the normal state and proliferation of cells. For instance, when the serum concentration in the cell growth medium was reduced to 2%, the viability of U87 and U373 glioblastoma cells decreased by approximately 40% at 68 h compared to cells cultured under 10% FBS conditions (Morais et al., 2022). Moreover, compared to 10% FBS, 2% FBS in the growth medium inhibits the cell viability and DNA replication of human primary adipocytes (Qian et al., 2021). Porcine mammary cells (PMCs) also exhibited a greater tendency towards senescence in low-serum medium (1% FBS) compared to 10% FBS medium (Huang et al., 2023). Overall, medium with FBS concentrations below 5% are inadequate for maintaining cell state. While 5% FBS medium without any cytokine addition can maintain the normal state of cells, it does not effectively promote cell proliferation.

In recent years, cytokines and growth factors have emerged as promising serum alternatives due to their defined nature and specific regulatory effects (Saxton et al., 2023). However, inherent limitations persist regarding the individual applications of these factors. While EGF promotes stem cell self-renewal through the EGFR/Ras-MAPK signaling pathway (Chen et al., 2021; Haraguchi et al., 2025), and bFGF maintains pluripotency via the PI3K-Akt/PLCγ-PKC pathways, these singular approaches fail to replicate the complex signaling networks necessary for comprehensive cellular regulation (Guo et al., 2012; Ishikawa et al., 2012; Choi et al., 2019; Jardé et al., 2020). This limitation is particularly critical for muscle stem cells, which require coordinated control over proliferation, maintenance of stemness, and differentiation. Similarly, small molecules such as HDAC inhibitors can modulate specific cellular processes but lack the pleiotropic effects characteristic of cytokines (Li et al., 2014; Palla et al., 2021). To address these constraints, we propose a synergistic strategy that combines cytokines and small molecules to mimic the pleiotropic effects of serum while ensuring cost-effectiveness (Wang et al., 2017). Furthermore, innovative approaches are being explored to enhance the sustainability and efficiency of low-serum systems, including the application of artificial intelligence to optimize reduced-serum formulations for cultivated meat (Nikkhah et al., 2023). For example, Beyond myogenesis, low-serum conditions (5% FBS) enhance the osteogenic potential of mesenchymal stem cells (MSCs) by mimicking physiological niches, while hypoxic low-serum cultures improve isolation efficiency and osteogenic differentiation in bone marrow-derived stem cells (Binder et al., 2015; Suehiro et al., 2017). In addition, the cell proliferation level of fish primary myoblasts in the 5% FBS group, which was supplemented with Auxenochlorella pyrenoidosa protein extract (APE) group, was significantly higher than that in the 10% FBS group (Dong et al., 2024). Additionally, the bovine myoblasts exhibited significantly enhanced cell proliferation level under low serum conditions (10% FBS) when supplemented with the Grifola frondosa extract (GFE) compared to those in the 20% FBS group (Yu et al., 2024). Despite these advances, existing serum-free or low-serum media often necessitate complex formulations and fail to sustain long-term proliferation and differentiation potential across species, particularly for porcine cells. Moreover, these systems overlook transfection efficiency, which is a critical requirement for genetic engineering in cellular agriculture. This highlights the need for a universally adaptable, transfection-optimized culture system that addresses both biological and practical constraints.

In this study, we aimed to bridge the existing gap by developing an optimized factor cocktail for porcine cell culture applications. Through systematic screening of cytokines and growth factors, we identified an optimized proliferation synergy factor cocktail (PSFC) that compensates for serum reduction while maintaining myogenic differentiation potential. This system demonstrates broad compatibility across multiple cell types, exhibiting enhanced transfection efficiency with a 12%–23% improvement across various cell lines. Furthermore, RNA sequencing analysis confirmed that cultures maintained in low-serum conditions (5% FBS) preserved native transcriptional profiles, thereby validating the system’s ability to sustain physiological cell states. Notably, this system reduced serum dependence by 75% and reduce the cost of cell culture. By balancing biological performance with economic viability, this study addresses a significant gap in low-serum culture technology for PSCs and PKFs, presenting a scalable and cost-effective solution for cell expansion applicable to biomedical research, therapeutic development, and biomanufacturing.

## 2 Materials and methods

### 2.1 Animals

All procedures involving animals were carried out in accordance with the guidelines of good laboratory practice and animals were provided with nutritious food and adequate water. Animal feeding and testing were performed in accordance with the National Research Council Guidelines for the Care and Use of Laboratory Animals and approved by the Institutional Animal Care and Use Committee of Huazhong Agricultural University. Piglets were slaughtered according to the standard procedures of the guidelines in the Regulations of the Standing Committee of the People’s Congress of Hubei Province (Hubei Province, China, HZAUSW-2017-008). All pigs were obtained from the experimental pig farm of Huazhong Agricultural University.

### 2.2 Cell isolation and culture

#### 2.2.1 Primary cell isolation

Porcine skeletal muscle satellite cells (PSCs) were isolated from three 3-day-old Large White piglets. All the piglets are boars. The PSCs were isolated and cultured according to previously established protocols (Lv et al., 2020). In brief, porcine skeletal muscle was minced and digested using 2 mg/mL collagenase type I (Sigma-Aldrich, USA, 0130). The digestion was terminated by adding RPMI 1640 medium supplemented with 20% fetal bovine serum (FBS; Gibco, United States). The digests were then filtered through 100 μm, 70 μm, and 40 μm cell strainers. The phosphate-buffered saline (PBS) used for cell isolation contained 2% penicillin-streptomycin. The cells were cultured in growth medium (RPMI 1640 supplemented with 20% FBS, 1% non-essential amino acids, 1% Glutamax, and 2% penicillin-streptomycin) on collagen-coated cell culture plates at 37 °C in a 5% CO2 atmosphere. The cells were maintained *in vitro* with medium changes every 2–3 days and were passaged upon reaching 60% confluence, limiting expansion to passage 3 for subsequent sorting. To further purify the PSCs, a suspension in FACS buffer was subjected to antibody staining. The staining cocktail, applied for 30–45 min on ice shielded from light, comprised APC-labeled anti-human CD29 antibody (BioLegend, 303008, TS2/16, 1:40), PE-Cy7-labeled anti-human CD56 (BD, 335826, NCAM16.2, 1:40), FITC-labeled anti-sheep CD31 (BIO-RAD, MCA1097F, CO.3E1D4, 1:40), and FITC-labeled anti-sheep CD45 (BIO-RAD, MCA2220F, 1.11.32, 1:40). Following this incubation, the cells were washed twice with cold 1× PBS and then reconstituted in 1× PBS containing 2% FBS. The final isolation of the target F-PSC population was achieved using a Sony SH800S Cell Sorter.

Mouse skeletal muscle satellite cells (SSCs) were isolated from ten 3-week-old C57BL mice using the same protocol as for PSCs. All the mice are male mice.

Porcine kidney fibroblasts (PKFs) were isolated from piglets aged 1–3 days. The kidney tissue was minced, washed three times with PBS, and digested in a 1:2 enzyme mix of Trypsin and Collagenase I (with a tissue-to-enzyme ratio of 1:1) for 1–2 h at 37 °C in a 5% CO_2_ atmosphere. The resulting digest was filtered through 100 μm and 70 μm strainers, and the fibroblasts were subsequently seeded in culture dishes and cryopreserved the following day. PKFs were routinely cultured in growth medium composed of Dulbecco’s Modified Eagle Medium (DMEM; Gibco), supplemented with 10% fetal bovine serum (FBS; Gibco), at 37 °C in a humidified atmosphere containing 5% CO_2_.

#### 2.2.2 Cell line culture

C2C12 myoblasts were routinely cultured in growth medium composed of DMEM, supplemented with 10% FBS, at 37 °C in a humidified atmosphere containing 5% CO_2_. For myogenic differentiation, the cells were switched to DMEM supplemented with 2% horse serum (HS; Gibco, USA) upon reaching 80%–90% confluence.

HEK293T cells were cultured in high-glucose DMEM supplemented with 10% FBS and 1% penicillin-streptomycin (100 U/mL penicillin, 100 μg/mL streptomycin) at 37 °C in a humidified incubator with 5% CO_2_. For subculturing, confluent cells were washed with PBS, detached using 0.25% Trypsin-EDTA for 2–3 min at 37 °C, and neutralized with complete medium. Following centrifugation at 300 rpm for 5 min, the cells were reseeded at a split ratio of 1:3 in fresh medium. Routine passaging was conducted every 2–3 days to maintain cell confluence below 80%, with cells prepared for transfection experiments at 60%–70% confluence 24 h prior to use.

### 2.3 Experimental treatment

The dry powders of IGF-1 (Aladdin, #P05019), TGF-β (Aladdin, #P01137), IL-6 (GenScript, #Z03034-10), GM-CSF (GenScript, #Z02974-10), and G-CSF (GenScript, #Z05361-100) were dissolved in a medium containing 10% FBS, while the dry powders of VC and bFGF were dissolved in PBS. Riboflavin was dissolved in dimethyl sulfoxide (DMSO), ensuring that the final DMSO concentration in the culture medium did not exceed 0.1% (Nishimura et al., 2008; Srikuea and Suhatcho, 2018; Horcharoensuk, et al., 2022). The resulting stock solution was then added to the culture medium used for cell culture to achieve the desired concentrations. In certain experiments, cells were co-treated with multiple substances. The control medium for culturing PSCs and MSCs consisted of RPMI Medium 1640 supplemented with 20% FBS, 1% non-essential amino acids, 1% Glutamax, and 2% penicillin-streptomycin. Conversely, the control medium for culturing C2C12 and PKFs was composed of DMEM supplemented with 10% FBS and 1% penicillin-streptomycin.

The combination subtraction method was employed for screening combinations of substances, allowing for the elimination of less important components. A positive control was established by including all substances, while the experimental group involved the sequential removal of one substance at a time. The proliferation activities of each group were then compared.

### 2.4 EdU cell proliferation staining

Cell proliferation activity was assessed using the EdU Cell Proliferation Detection Kit (Beyotime, China, C0071S), following the manufacturer’s protocol. Briefly, cells were seeded in a 6-well plate at a density of approximately 7–9 × 10^4^ cells and incubated for 24 h. Subsequently, 10 μM EdU was added, and cells were incubated for an additional 2 h. The cells were then fixed with 4% paraformaldehyde, permeabilized with PBS containing 0.3% Triton X-100, and subjected to a Click reaction. Nuclear staining was performed using Hoechst 33342. Images were acquired using a fluorescence microscope (IX51-A21PH, Olympus, Tokyo, Japan).

### 2.5 Cell viability assay

Cell viability was measured using the Cell Counting Kit-8 (CCK-8) (Beyotime, China, C0038), in accordance with the manufacturer’s instructions. PSCs were plated in 96-well plates at a density of 0.5 × 10^4^ cells per well and incubated at 37 °C with 5% CO_2_ for 24 h. Two treatment groups were established: one with 20% FBS and the other with 20% FBS supplemented with cytokines. Measurements were taken at 24 h, 48 h, and 72 h post-treatment. To assess viability, 10 μL of CCK-8 solution was added to each well and incubated for 1 h. The absorbance was measured at 450 nm using a Spark Multimode Microplate Reader (Tecan, Switzerland).

### 2.6 Cellular immunofluorescence

Cellular immunofluorescence staining was conducted following established protocols in the literature (Lv et al., 2020). The primary antibodies used were MyHC (sc-376157; 1:0; Santa Cruz Biotechnology, Dallas, Texas, United States) and MyoG (sc-12732; 1:0; Santa Cruz Biotechnology, Dallas, Texas, USA), along with the secondary antibody A0521 (Goat anti-mouse CY3, Beyotime Biotechnology, Shanghai, China). Observations were made using a fluorescence microscope. In accordance with the manufacturer’s instructions, Actin-Tracker Red-555 (Beyotime, China, C2203S) was utilized to stain cellular actin. Cells were fixed using an immunostaining fixative (Beyotime, China, P0098) for 30 min, followed by washing with PBS containing 0.1% Triton X-100. The Actin-Tracker Red was diluted with the immunofluorescence secondary antibody dilution solution (Beyotime, China, P0108) at a ratio of 1:200 and incubated in the dark for 1 h. After incubation, the cells were washed again with PBS containing 0.1% Triton X-100, and images were captured using a fluorescence microscope.

### 2.7 Total RNA preparation and real-time quantitative PCR (RT-qPCR)

Total RNA from cells was isolated according to the manufacturer’s instructions using Trizol reagent (Invitrogen, Carlsbad, CA, United States). The concentration of the isolated RNA was determined using a NanoDrop 2000 spectrophotometer. Complementary DNA (cDNA) was synthesized using the HiScript III RT SuperMix for qPCR with gDNA wiper (Novozan, R323-01) kit. RT-qPCR analysis was conducted on the Applied Biosystems StepOnePlus real-time fluorescence quantitative PCR system. The relative RNA expression levels were calculated using the Ct (2^−ΔΔCT^) method. The complete primer sequences utilized for RT-qPCR analysis are detailed in Table 1.

**TABLE 1:** RT-qPCR primers used in this paper.

Gene	Species	Primer-F	Primer-R
*KI-67*	pig	GGTGACTTGAAAACGGACGC	TGGGATTTTCGGCTCCATCC
*P53*	pig	TGACTGTACCACCATCCACTAC	AAACACGCACCTCAAAGC
*CDK1*	pig	ACACGTTGTATCAAGAACAGATAGT	AGGTTGTTACAGTGGAATCTACA
*MYOD*	pig	AAGTCAACGAGGCCTTCGAG	GGGGGCCGCTATAATCCATC
*MYOG*	pig	AGGCTACGAGCGGACTGA	GCAGGGTGCTCCTCTTCA
*MYHC*	pig	GAAGAGTACGCCAAGGGGAAA	GTGTACCGGTCCTTGAGGTT
*ESM1*	pig	CTCGGAGAAACCTGCTACCG	TTGCATTCCATCCCGAAGGT
*MT1A*	pig	CTCCACTCATGGACCCCAAC	AGGAGCAGCAGCTCTTCTTG
*MKI67*	pig	ACAACAGGAGGAGGAAGTGCT	TTGACCTAGACGCGGGGAT
*CCND1*	pig	GACCGCTTCCTGTCCCTG	GTGGCACAGAGGGCGACGA
*HMOX1*	pig	GACATGGCCTTCTGGTATGGG	CATGTAGCGGGTGTAGGCGT
*PCNA*	pig	TGCAGATGTACCCCTTGTTGT	CATCTTCGATCTTGGGAGCCA
*CRISPLD2*	pig	GCTGGAGCTGTTCAAGATGG	TCCAGGTCCACATCCTTCTG
*HSPA2*	pig	CAGGACCTGAAGGTGCTGAT	GTCCTTGGGGTCATCTTCAG
*ITGA3*	pig	CTGGGCTTCCTCTGTGACTC	GCCATTGTCACAGGGTCTTC
*AREG*	pig	TGCTGGCTCTGTGTTCTGTG	CAGGGTCCACTGGTTCTCTT
*ADAM8*	pig	GCCCTGTGTTCTGTGGAGAT	GGGTAGGCACAGTGATGAGC
*SEMA7A*	pig	CCTGGGACTGCTACCTGTTC	GCTGGTAGGGTTGGTGTTGT
*GAPDH*	pig	TGCTCCTCCCCGTTCGAC	ATGCGGCCAAATCCGTTC
*MyOD*	mouse	GGCTGCCCAAGGTGGAAATC	TGCGTCTGAGTCACCGCTGTAG
*MyOG*	mouse	ATGAGACATCCCCCTACTTCTACCA	GTCCCCAGCCCCTTATCTTCC
*MyHC*	mouse	CAAGTCATCGGTGTTTGTGG	TGTCGTACTTGGGCGGGTTC
*Scn5a*	mouse	ATGGCAAACTTCCTGTTACCTC	CCACGGGCTTGTTTTTCAGC
*Il33*	mouse	TCCAACTCCAAGATTTCCCCG	CATGCAGTAGACATGGCAGAA
*Acta1*	mouse	CCCAAAGCTAACCGGGAGAAG	CCAGAATCCAACACGATGCC
*Id3*	mouse	CTGTCGGAACGTAGCCTGG	GTGGTTCATGTCGTCCAAGAG
*Spp1*	mouse	AGCAAGAAACTTTCCAAGCAA	GTGATTCGTAGATTCATCCG
*HGF*	mouse	CTGCAGATGAGTGTGCCAAC	CCAGTAGCATCGTTTTCTTGACT
*β-actin*	mouse	TCTGGCACCACACCTTCTA	AAGGTCTCGAACATGATCTG

### 2.8 Western blotting

Cell proteins were isolated using radioimmunoprecipitation assay (RIPA) buffer supplemented with 1% phenylmethylsulfonyl fluoride (Beyotime Biotechnology, Shanghai, China). Western blotting was performed as previously described. The antibodies used included MyoG (sc-12732; 1:0; Santa Cruz Biotechnology, Dallas, Texas, United States), MyHC (sc-376157; Santa Cruz Biotechnology, Dallas, Texas, United States), β-actin (sc-4777; Santa Cruz Biotechnology, Dallas, Texas, United States), Ki67 (ab16667; 1:1000; Abcam, Cambridge, UK), and anti-mouse IgG secondary antibodies (1:3000; Santa Cruz Biotechnology, Dallas, Texas, United States). All protein levels were normalized to the housekeeping protein β-actin, and the western blotting bands were quantified using a standardized grayscale measurement scheme through ImageJ software.

### 2.9 Cell count

Upon reaching the required growth time, a cell suspension was prepared. The cell counting plate was covered with a cover slip, and the cell suspension was gently dropped between the cover slip and the cell counting plate from the edge. A 10× eyepiece was used to count the cells in the four large squares under an inverted microscope, adhering to the principle of counting the top but not the bottom, and counting the left but not the right.

### 2.10 RNA-seq and bioinformatics analysis

In the control group and the 5% serum group, C2C12 cells were cultured for proliferation. After the cell density reached 80%, 2%HS was replaced for differentiation for 5 days. Total RNA was isolated using Trizol (ThermoFisher, 10296028). Genomic DNA was removed using Turbo DNA-Free Kit (ThermoFisher, AM 1907). Total RNA integrity was determined using RNA 6000 Nano Kit (Agilent, 5067-1511). Libraries of amplified RNA were prepared in accordance with the Illumina protocol. The clustering of the index-coded samples was performed using TruSeq PE Cluster Kit v3-cBot-HS (Illumia). The library preparations were sequenced and 150 bp paired-end reads were generated (Novogene, Beijing, China). After adaptor trimming and low-quality reads removing by Trim-Galore (v 0.6.6), the high-quality clean reads were aligned to the reference genome (UCSC mm10) with Hisat2 (v2.2.1). The numbers of reads mapped to each gene were counted using FeatureCounts (v2.0.1). Differential expression analysis of the genes was performed using the R package DESeq2 (v1.28.1). Genes with an adjusted p value (padj) < 0.05 and |fold change|> 1.5 were assigned as differentially expressed genes (DEGs). This threshold was selected to capture the subtle transcriptional alterations expected from the serum reduction treatment, while maintaining statistical rigor through FDR control. Gene ontology (GO) and Kyoto Encyclopedia of Genes and Genomes (KEGG) enrichment analyses of DEGs were performed using the R package Cluster Profler (v3.0.4).

### 2.11 Plasmid construction and cell transfection

The experimental procedure commenced with the single-enzyme digestion of the PX458 vector using Bbs I restriction endonuclease. This was followed by ligation with annealed primers employing T4 DNA ligase and subsequent transformation for plasmid amplification. The specific primers related to the genes are presented in Table 2. The resultant plasmids were utilized to transfect cells cultured under various media conditions (20% FBS, 20% FBS-PSFC, and 5% FBS-PSFC) at 50% confluency, using 5 μg of plasmid DNA complexed with Lipofectamine 2000, in accordance with the manufacturer’s protocol. Transfection efficiency was quantitatively assessed 24–48 h post-transfection through fluorescence intensity measurement utilizing a full-spectrum flow cytometer (Cytek Biosciences, USA), accompanied by parallel qualitative evaluation via fluorescence microscopy.

**TABLE 2:** Oligonucleotide primers used in this paper.

Gene	Species	Primer-F	Primer-R
*CD163*	pig	GGTGACTTGAAAACGGACGC	TGGGATTTTCGGCTCCATCC
*FHL3*	pig	TGACTGTACCACCATCCACTAC	AAACACGCACCTCAAAGC
*CD163*	mouse	ACACGTTGTATCAAGAACAGATAGT	AGGTTGTTACAGTGGAATCTACA
*FHL3*	mouse	TGTACGGCCGCAAATACATCCAGA	TCTGGATGTATTTGCGGCCGTACA
*VEGF*	human	ATGAGACATCCCCCTACTTCTACCA	GTCCCCAGCCCCTTATCTTCC
*RUNX1*	human	TCTGGCACCACACCTTCTA	AAGGTCTCGAACATGATCTG

### 2.12 3D culture mold preparation and cell culture

The commercially available curing rings utilized were silicone-based circular molds (EngineeringForLife, Catalog Number: EFL-SCR-3D-48-1-12), measuring 1 cm in diameter and 0.5 cm in height. Prior to use, the microcolumn molds were sterilized through sequential washing with distilled water, autoclaving, immersion in 75% ethanol for 15 min, and exposure to UV irradiation for 30 min. The water bath was preheated to 70 °C for the preparation of liquid GelMA hydrogel (SUNP BIOTECH, SP-BI-G01-2). A 4% (w/v) hydrogel solution was prepared by dissolving 500 mg of GelMA powder in 12.5 mL of 10% FBS proliferation medium, followed by complete dissolution via water bath heating. The liquid hydrogel was then sterile-filtered through a 0.22 μm membrane and stored at 4 °C. After cell counting, the cell-hydrogel mixture was uniformly inoculated into the curing rings. The resulting 3D constructs were transferred to culture dishes containing proliferation medium (RPMI-1640 with 20% FBS), with hydrogel detachment performed after 2–3 days of culture. Following 2 days of proliferation in GelMA hydrogels, PSCs were differentiated for 8 days in DMEM supplemented with 2% HS. Myotube formation was subsequently verified through observation using bright-field microscopy (Dutta et al., 2022; Hong et al., 2023; Niu et al., 2024).

### 2.13 Statistical analysis

All data were expressed as mean ± standard deviation (n = 3). Statistical analysis was performed using GraphPad Prism software. For comparisons between two groups, both paired and unpaired Student's t-tests were employed. For analyses involving three or more groups, one-way analysis of variance (ANOVA) with Tukey’s multiple comparison tests was used for statistical analyses. *P* value less than 0.05 was considered statistically significant. *P < 0.05, **P < 0.01, ***P < 0.001, ****P < 0.0001.

## 3 Results

### 3.1 Chemically defined proliferation screening of porcine muscle satellite cells

**FIGURE 1:**
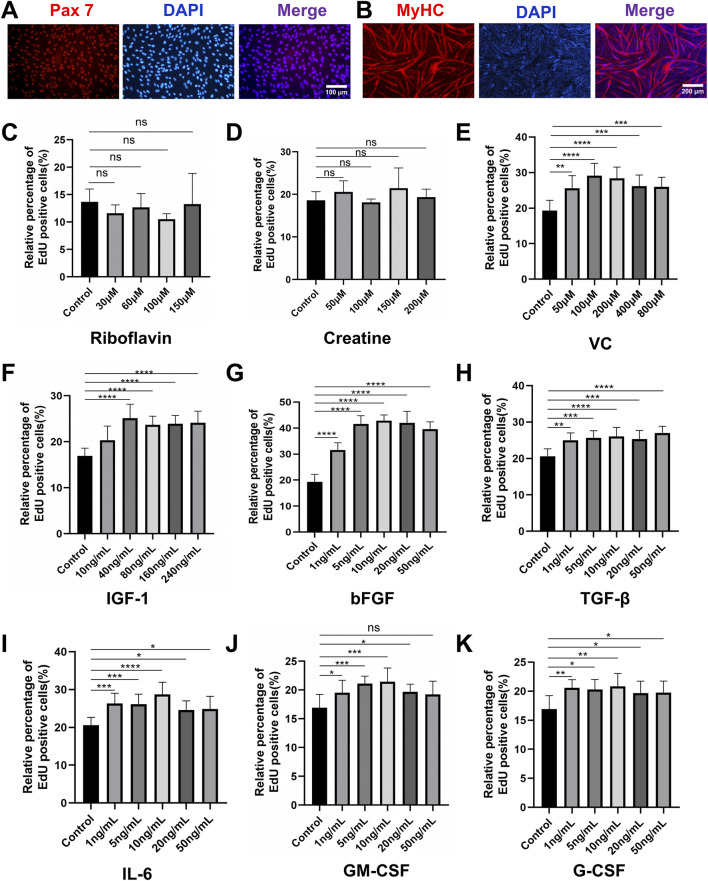
Characterization and proliferation screening of porcine muscle satellite cells (PSCs). **(A)** Immunofluorescence staining confirmed high expression of the canonical satellite cell marker Pax7 in isolated cells. **(B)** Immunofluorescence staining confirmed that PSCs differentiated in 2% HS for 4 days formed multinucleated myotubes and exhibited robust MyHC expression. **(C,D)** EdU proliferation assay after 24-h treatment with small molecules and cytokines. Neither riboflavin nor creatine treatment showed significant mitogenic effects compared to controls. **(E–K)** Dose-dependent proliferation enhancement was observed for **(E)** VC (optimal concentration: 100 μM), **(F)** IGF-1 (40 ng/mL), **(G)** bFGF (10 ng/mL), **(H)** TGF-β (5 ng/mL), **(I)** IL-6 (10 ng/mL), **(J)** GM-CSF (10 ng/mL), and **(K)** G-CSF (10 ng/mL). Data are shown as mean ± SD, n = 3. *P < 0.05, **P < 0.01, ***P < 0.001, ****P < 0.0001.

Primary PSCs were isolated from the longissimus dorsi and leg muscles of 1-3-day-old piglets via a two-step enzymatic digestion protocol (Li et al., 2022). To minimize fibroblast contamination, cells were subjected to differential plating on Matrigel, followed by fluorescence-activated cell sorting (FACS) using CD31^-^/CD45^-^ and CD56^+^/CD29^+^ markers. Immunofluorescence staining confirmed high Pax7 expression, validating the satellite cell identity (Figure 1A). The isolated PSCs exhibited robust myogenic potential, evidenced by multinucleated myotube formation and upregulated myosin heavy chain (MyHC) expression upon differentiation in 2% horse serum (HS) (Figure 1B). To establish a chemically defined proliferation system, we systematically evaluated nine small molecules and cytokines for their effects on PSCs proliferation. These factors included riboflavin, creatine, ascorbic acid (vitamin C, VC), insulin-like growth factor-1 (IGF-1), basic fibroblast growth factor (bFGF), transforming growth factor-β (TGF-β), interleukin-6 (IL-6), granulocyte-macrophage colony-stimulating factor (GM-CSF), and granulocyte colony-stimulating factor (G-CSF). EdU assays after 24 h revealed that riboflavin and creatine had no significant proliferative effect (Figures 1C,D; Supplementary Figure S1). In contrast, seven factors (VC, IGF-1, bFGF, TGF-β, IL-6, GM-CSF, and G-CSF) demonstrated dose-dependent enhancements in proliferation (Figures 1E–K; Supplementary Figure S1). The optimal concentrations were determined to be 100 μM for VC, 40 ng/mL for IGF-1, 10 ng/mL for bFGF, 5 ng/mL for TGF-β, and 10 ng/mL for IL-6, GM-CSF, and G-CSF. These concentrations were subsequently employed in combinatorial experiments based on their efficacy and practical considerations.

### 3.2 Optimization of a minimal growth factor for cost-effective expansion of PSCs

**FIGURE 2:**
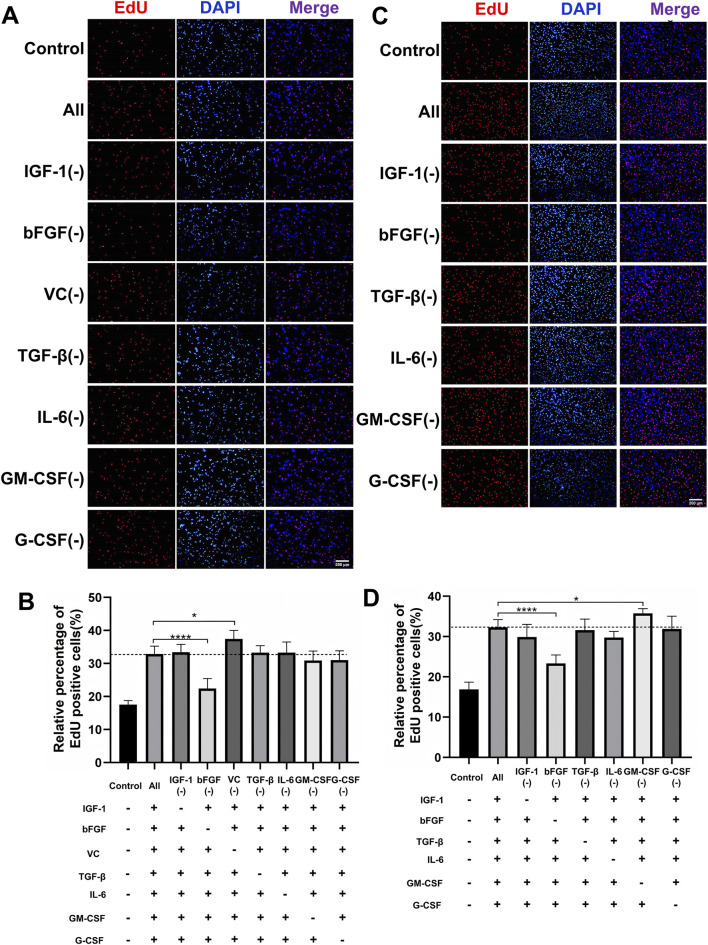
Systematic optimization of a minimal growth factor formulation for PSCs proliferation. **(A,B)** Proliferative effects of the initial seven-factor cocktail on PSCs assessed by EdU incorporation assay. The initial seven factors were evaluated at their predetermined optimal concentrations: IGF-1 (40 ng/mL), bFGF (10 ng/mL), VC (100 μM), TGF-β (5 ng/mL), IL-6 (10 ng/mL), GM-CSF (10 ng/mL), and G-CSF (10 ng/mL). **(C,D)** Experimental design evaluating six growth factors at optimized concentrations: IGF-1 (40 ng/mL), bFGF (10 ng/mL), TGF-β (5 ng/mL), IL-6 (10 ng/mL), GM-CSF (10 ng/mL), and G-CSF (10 ng/mL). Quantitative assessment of proliferation capacity by EdU incorporation assay. The GM-CSF (−) group showed significantly enhanced proliferation compared to the complete six-factor control. Data are shown as mean ± SD, n = 3. *P < 0.05, ****P < 0.0001. “+” indicates inclusion of the component; “−” indicates exclusion of the component, blank control groups received no additional components.

To develop a scalable and cost-effective proliferation system for PSCs, we adopted a combinatorial elimination approach to identify the minimal growth factor requirements. Given that overly complex serum-free formulations can hinder scalability, we aimed to eliminate non-essential components while maintaining robust proliferation. Through two iterative screening rounds, we first evaluated seven factors (IGF-1, bFGF, TGF-β, IL-6, GM-CSF, G-CSF, and VC) at their previously optimized concentrations (Figure 2A). EdU assays indicated that the omission of VC significantly increased the proportion of EdU^+^ cells compared to the complete cocktail (Figure 2B), suggesting that VC may inhibit the synergistic activity of the other factors. Further screening of the remaining six factors revealed that the exclusion of GM-CSF further enhanced cell proliferation, as evidenced by a higher percentage of EdU^+^ cells in the GM-CSF(−) group (Figures 2C,D). After two rounds of systematic screening, we developed an optimized five-factor formulation comprising IGF-1, bFGF, TGF-β, IL-6, and G-CSF, hereafter referred to as the proliferation synergy factor cocktail (PSFC), which maximizes the efficiency of PSCs proliferation.

**FIGURE 3:**
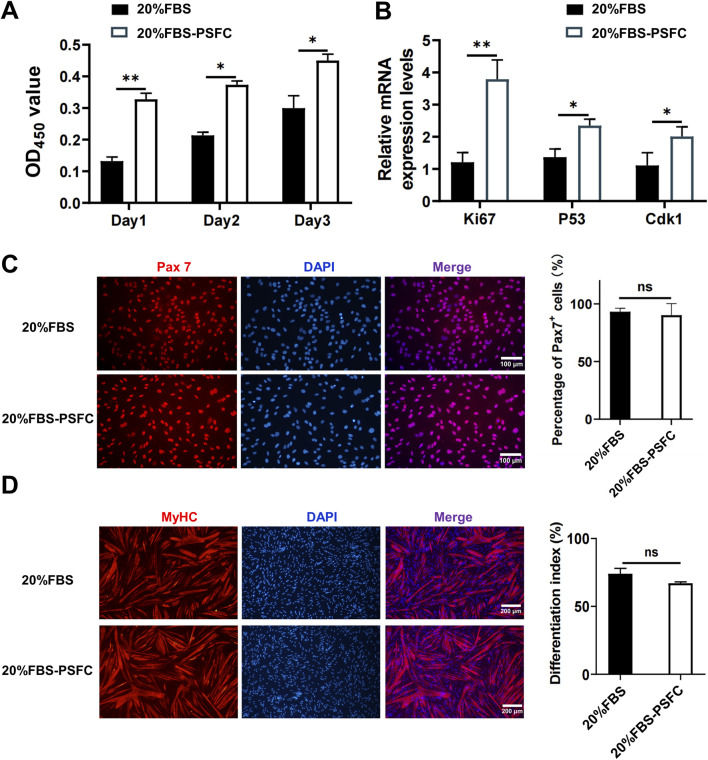
Validation of the proliferation synergy factor cocktail (PSFC) for enhanced expansion and maintenance of stemness in PSCs. **(A)** CCK-8 assays were conducted to compare the cell expansion of PSCs cultured in 20% FBS as the basal control and 20% FBS-PSFC. The results indicated that 20% FBS-PSFC significantly promoted cell expansion compared to 20% FBS. **(B)** RT-qPCR analysis of proliferation-associated genes (*Ki67*, *MyoD*, *p53*, and *Cdk1*) revealed significant upregulation in 20% FBS-PSFC cultured PSCs compared to 20% FBS. **(C)** Immunofluorescence analysis demonstrated comparable Pax7^+^ cell fractions between 20% FBS and 20% FBS-PSFC groups, confirming maintained stem cell properties. **(D)** Post-induction MyHC immunostaining showed no significant differences between groups, indicating preserved differentiation potential in PSFC-treated PSCs. Data are shown as mean ± SD, n = 3. *P < 0.05, **P < 0.01. ns indicates statistical non-significance.

To validate the long-term effects of PSFC, we conducted serial passaging assays under two conditions: a basal control consisting of 20% FBS and an optimized condition of 20% FBS supplemented with PSFC (20% FBS-PSFC). CCK-8 assays demonstrated that 20% FBS-PSFC significantly enhanced cell expansion compared to 20% FBS (Figure 3A). Consistent with these findings, RT-qPCR analysis revealed a significant upregulation of proliferation-associated genes, including *Ki67*, *MyoD*, *P53*, and *Cdk1* in 20% FBS-PSFC (Figure 3B). Immunostaining confirmed that PSFC maintained the stemness of PSCs with the Pax7^+^ cell fractions remaining comparable to 20% FBS, while MyHC expression exhibited no significant changes following myogenic induction (Figures 3C,D). Collectively, these findings establish PSFC as an effective minimal formulation that enhances PSCs proliferation beyond standard FBS conditions while preserving stemness and differentiation potential.

### 3.3 5%FBS-PSFC sustains PSCs proliferation and differentiation capacity

**FIGURE 4:**
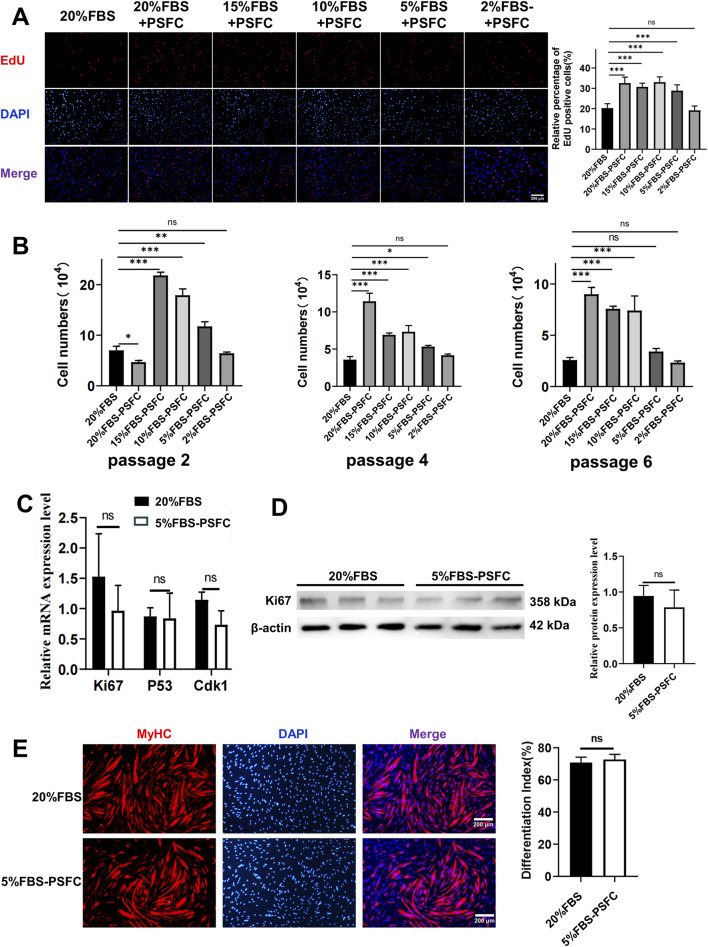
5%FBS-PSFC maintains proliferation and differentiation capacity of PSCs under reduced serum conditions. **(A)** EdU assays were conducted after 24 h of culture in varying serum concentrations supplemented with PSFC. **(B)** Cell counting during serial passaging (P2-P6) demonstrated that 5%FBS-PSFC sustained proliferation comparable to 20%FBS, whereas 2%FBS-PSFC was unable to maintain expansion beyond P2. **(C)** RT-qPCR analysis revealed equivalent mRNA expression of *Ki67*, *p53*, and *Cdk1* in 5%FBS-PSFC compared to 20%FBS. **(D)** Western blot analysis confirmed similar Ki67 protein levels. **(E)** Immunofluorescence of MyHC indicated comparable myotube formation efficiency between the 5%FBS-PSFC and 20%FBS groups. Data are shown as mean ± SD, n = 3. *P < 0.05, **P < 0.01, ***P < 0.001, ****P < 0.0001. ns indicates statistical non-significance.

Using the optimized PSFC formulation, we next evaluated the minimal serum requirements for PSC expansion by testing a series of FBS gradients: 20%, 15%, 10%, 5%, and 2% FBS-PSFC (Table 3). Initial EdU assays conducted at 24 h confirmed that PSFC maintained robust proliferative activity even at reduced serum concentrations, with only the 2% FBS-PSFC group exhibiting baseline-level proliferation (Figure 4A). To further assess long-term proliferation, we conducted serial passaging (P2, P4, and P6) followed by morphological analysis. Bright-field imaging demonstrated that all experimental groups maintained a spindle-shaped morphology up to P6. Notably, the 20% FBS group exhibited characteristic senescent morphology by passage 6, including cellular flattening and vacuolization, whereas the treatment groups maintained their pristine cytoarchitecture (Supplementary Figure S2). Cell counting revealed that cultures supplemented with PSFC in 10%–20% FBS exhibited significantly higher cell yields compared to serum-only controls (Figure 4B). Strikingly, the 5% FBS-PSFC group exhibited proliferation rates comparable to those observed in conventional 20% FBS cultures, indicating that the PSFC effectively compensated for the 75% reduction in serum. In contrast, the 2% FBS-PSFC cultures demonstrated a progressive decline in proliferation after P2, failing to reach confluency by P6. Therefore, we established 5% FBS as the minimal serum threshold necessary for sustained expansion. RT-qPCR analysis confirmed that key cell cycle regulators (Ki67, p53, and Cdk1) were expressed at comparable levels in PSCs cultured in 5% FBS-PSFC and 20% FBS. Western blot analysis validated these findings at the protein level, with consistent Ki67 expression indicating that the low-serum formulation effectively maintained normal cell cycle progression (Figures 4C,D). Given the critical importance of differentiation capacity, we subsequently investigated whether serum reduction during proliferation affected subsequent myogenic potential. Following expansion in either 5%FBS-PSFC or 20%FBS conditions, PSCs were induced to differentiate in a medium containing 2% HS. After 48 h, both groups efficiently formed multinucleated myotubes. Immunofluorescence analysis revealed comparable MyHC expression, confirming that low-serum culture did not impair differentiation (Figure 4E).

**TABLE 3:** Serum concentration optimization for PSCs expansion using PSFC formulation.

Medium component names	Cytokines	Serum concentration
20% FBS	None	20% FBS
20% FBS-PSFC	IGF-1 (40 ng/mL) + bFGF (10 ng/mL) + TGF-β (5 ng/mL) + IL-6 (10 ng/mL) + G-CSF (10 ng/mL)	20% FBS
15% FBS-PSFC		15% FBS
10% FBS-PSFC		10% FBS
5% FBS-PSFC		5% FBS
2% FBS-PSFC		2% FBS

### 3.4 5% FBS-PSFC system maintains proliferation and differentiation capacities across diverse cell types

**FIGURE 5:**
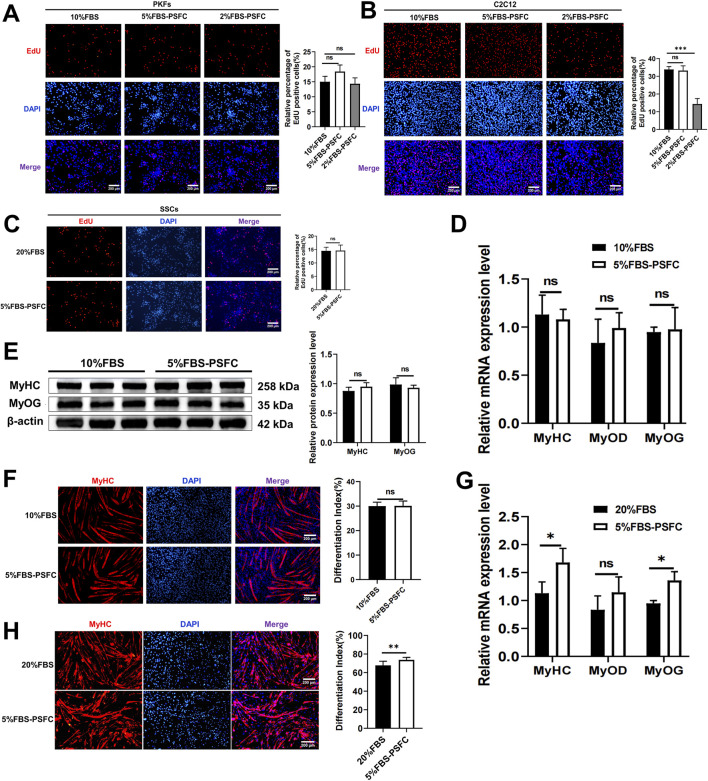
The proliferation and differentiation capabilities of various cell types were cross-species validated using the 5% FBS-PSFC platform. **(A–C)** EdU assay in three cell types under varying serum conditions. **(A)** PKFs cultured in 5% FBS-PSFC or 2% FBS-PSFC showed comparable proliferation rates to those in 10% FBS (control), as measured by EdU + cell percentage, suggesting that PKFs proliferation remains unaffected by reduced serum concentrations. **(B)** The percentage of EdU^+^ C2C12 cells cultured in conditions with 10% FBS (control), 5% FBS-PSFC, and 2% FBS-PSFC. Compared to the 10% FBS control group, the 2% FBS-PSFC group exhibited a significant reduction in cell proliferation, indicating that 2% serum is insufficient to sustain the growth of C2C12 myoblasts. **(C)** Quantification of EdU^+^ SSCs showed no significant difference in proliferation between 5% FBS-PSFC and 20% FBS (control) conditions, confirming that serum reduction does not impair SSC proliferation. **(D)** RT-qPCR showed no detectable differences in the mRNA expression of *MyoD*, *MyoG*, and *MyHC* when cultured in 5% FBS-PSFC. **(E)** Western blotting of differentiated C2C12 myoblasts showed that MyoG and MyHC protein levels unchanged compared to control group. **(F)** The quantification of MyHC immunofluorescence staining results showed no significant difference in the protein expression. **(G)** Quantitative RT-PCR analysis revealed significant increases in mRNA expression levels of *MyoG* and *MyHC* in SSCs cultured under 5% FBS-PSFC conditions compared to controls. **(H)** Immunofluorescence staining demonstrated elevated MyHC protein expression in SSCs cultured in 5% FBS-PSFC medium, confirming their myogenic differentiation potential. Data are shown as mean ± SD, n = 3. *P < 0.05, **P < 0.01, ***P < 0.001. ns indicates statistical non-significance.

To validate the universal applicability of our optimized PSFC culture platform, we assessed its performance across multiple cell types and species. Proliferation assays demonstrated consistent proliferative capacity in all tested models, including porcine kidney fibroblasts (PKFs), myoblasts (C2C12) and mouse skeletal muscle satellite cells (SSCs). Notably, all cell types exhibited similar proliferation rates in 5%FBS-PSFC culture compared to conventional FBS conditions (Figures 5A–C). Since PKFs and C2C12 are routinely cultured in 10% FBS, we further examined the effects of 2% FBS-PSFC. Notably, the proportion of EdU^+^ cells in PKFs showed no significant difference between the 5% FBS and 2% FBS treatments compared to the control group with 10% FBS (Figure 5A). This indicated that PKFs may be capable of proliferation in lower serum concentrations. In contrast, the proportion of EdU^+^ cells in the C2C12 exposed to 2% FBS-PSFC was significantly reduced compared to the control group (Figure 5B). This finding suggested that 2% serum is insufficient to sustain the proliferation of C2C12 myoblasts. Additionally, C2C12 myoblasts showed no detectable differences in the mRNA expression of *MyoD*, *MyoG*, and *MyHC* when cultured in 5% FBS-PSFC (Figure 5D). Similarly, MyHC protein expression and fusion indices remained unchanged compared to controls (Figures 5E,F). Strikingly, the low-serum system not only maintained but also enhanced myogenic differentiation in SSCs. SSCs cultured in 5%FBS-PSFC exhibited a significant upregulation of the differentiation markers *MyoG* and *MyHC* at the mRNA expression level, with immunofluorescence confirming an increase in MyHC^+^ myotube formation (Figures 5G,H). In summary, these findings indicated that our 5% FBS system supports robust proliferation and differentiation across various cell types and species, while simultaneously reducing serum requirements by 50%.

### 3.5 5% FBS-PSFC system significantly enhances transfection efficiency across diverse cell types

**FIGURE 6:**
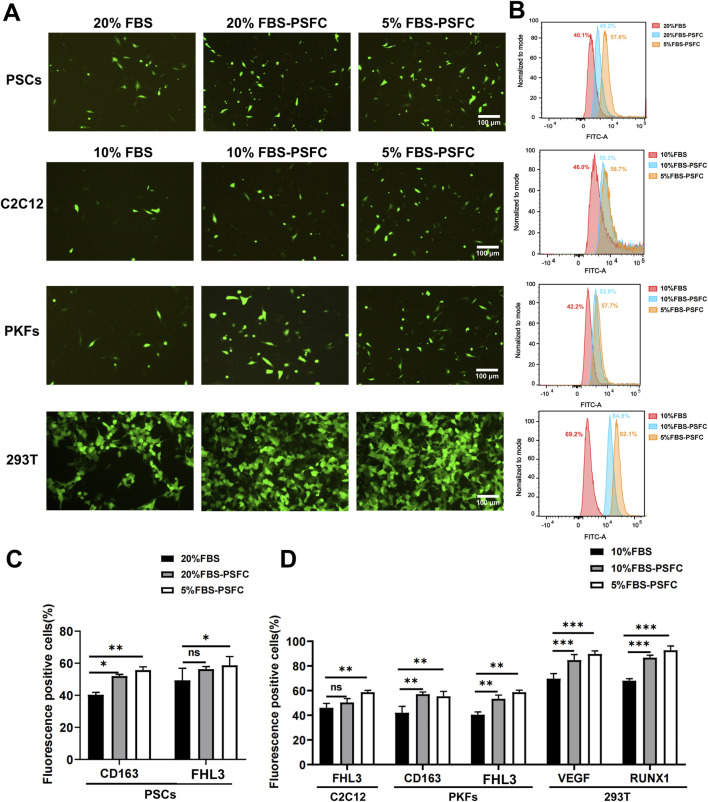
Significant enhancement of transfection efficiency in low-serum (5%FBS-PSFC) culture system **(A)** Representative fluorescence microscopy images demonstrating markedly improved GFP expression (green) across all tested cell types (PSCs, C2C12, PKFs, and HEK293T) under optimized culture conditions. **(B)** Quantitative flow cytometry profiles revealing distinct rightward shifts in fluorescence intensity distributions (FITC-A channel) for cells cultured in 5% FBS-PSFC, indicating significantly higher GFP expression levels. **(C)** Statistical analysis of PSC transfection efficiency showing that 5% FBS-PSFC achieved a remarkable 17.5% increase in GFP-positive cells compared to standard 20% FBS culture. **(D)** Comparative transfection performance across multiple cell lineages, with 5% FBS-PSFC consistently demonstrating superior efficiency: C2C12, PKFs, and HEK293T compared to their respective 10% FBS controls. The histogram overlays (normalized cell counts vs. fluorescence intensity) clearly demonstrate that serum reduction combined with PSFC synergistically enhances nucleic acid delivery efficiency across all cell types tested. Data are shown as mean ± SD, n = 3. *P < 0.05, **P < 0.01, ***P < 0.001. ns indicates statistical non-significance.

Given that high serum concentrations are known to inhibit the uptake of exogenous nucleic acids due to serum protein interference with transfection complexes, we hypothesized that a reduced serum environment (5% FBS) combined with PSFC would facilitate more efficient delivery (Zuris et al., 2015). To test this, we conducted systematic transfection comparisons across multiple cell types under varying culture conditions. PSCs were cultured under three conditions: conventional 20% FBS (control), 20% FBS-PSFC, and 5% FBS-PSFC. Similarly, C2C12 myoblasts, PKFs, and HEK293T cells were assessed under 10% FBS (control), 10% FBS-PSFC, and 5% FBS-PSFC. As demonstrated by fluorescence microscopy and flow cytometry analysis (Figures 6A,B), the 5% FBS-PSFC culture system significantly enhanced transfection efficiency across all tested cell types compared to conventional serum conditions. Quantitative analysis revealed cell type-specific improvements (Figures 6C,D). PSCs exhibited a 17.5% increase in GFP^+^ cells, while PKFs showed a 15.5% enhancement in transfection efficiency. C2C12 myoblasts demonstrated a 12.7% improvement. HEK293T cells achieved the most substantial gain, with a 22.9% increase in transfected cells. Notably, the improvement in transfection efficiency was consistently observed across multiple plasmid constructs, suggesting a generalized enhancement of nucleic acid delivery rather than vector-specific effects. The conditions with 20% or 10% FBS supplemented with PSFC exhibited intermediate efficiency compared to conventional cultures and those utilizing 5% FBS-PSFC. These findings indicate that both the reduction of serum and the optimization of the small molecule cocktail contribute to the observed effects. The 5% FBS-PSFC culture system not only sustains cell proliferation but also significantly enhances transfection efficiency, with an average improvement of 16.9% depending on the cell type. The consistent performance across various cell lineages—including primary cells, immortalized lines, myogenic cells, and non-myogenic cells—suggests the broad applicability of this system in genetic engineering and cell therapy applications.

### 3.6 5% FBS-PSFC enables cost-effective 3D culture and scalable cultured meat production

**FIGURE 7:**
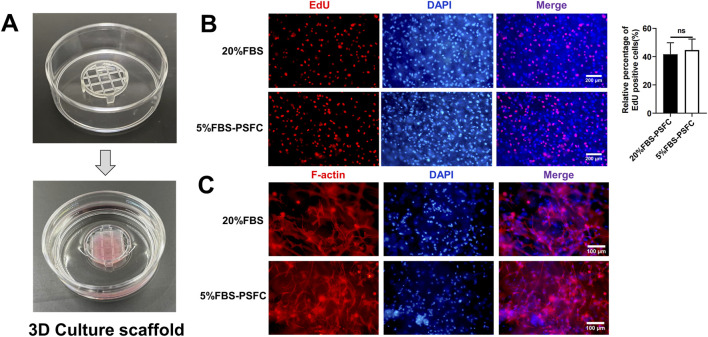
Evaluation of 5% FBS-PSFC system in 3D culture for cultured meat production. **(A)** Schematic of 3D cultivation using GelMA hydrogels. **(B)** Comparison of proliferation rates between culture conditions in 3D environment. **(C)** Immunofluorescence analysis of F-actin (red) and nuclei (DAPI, blue) in differentiated 3D cultures after 7 days. Data are shown as mean ± SD, n = 3. *P < 0.05, **P < 0.01, ***P < 0.001. ns indicates statistical non-significance.

Building on the advantages observed in two-dimensional (2D) culture, we next evaluated whether our 5% FBS-PSFC system could support three-dimensional (3D) cell growth, which is a critical requirement for scalable cultured meat production. Utilizing commercial solid scaffolds for 3D cultivation, PSCs encapsulated in gelatin methacryloyl (GelMA) hydrogels exhibited comparable proliferation rates under 5% FBS-PSFC conditions in contrast to 20% FBS controls (Figures 7A,B). Immunofluorescence analysis of F-actin confirmed a uniform distribution of cells and sustained cytoskeletal organization following 7 days of differentiation in 3D culture (Figure 7C). This demonstrates the system’s capability to preserve cellular integrity and differentiation potential under low-serum conditions. In addition, this optimized system reduced serum requirements by 75%, resulting in substantial cost savings of cell culture media (Table 4). The minimal PSFC formulation further decreased cytokine costs by 80%–90% compared to commercial serum-free supplements (such as StemPro™ MSC SFM XenoFree). These results position the 5% FBS-PSFC platform as both biologically effective and economically viable for scalable cultured meat production.

**TABLE 4:** Manufacturing cost of the PSFC.

Medium composition	Produced brand	20%FBS	5%FBS-PSFC
RPMI 1640	Gibco	$5.21/377.5 mL	$5.21/450 mL
FBS	HyClone	$82.76/100 mL	$20.69/25 mL
PS	Gibco	$3.94/10 mL	$3.94/10 mL
Glu	Gibco	$5.03/6.25 mL	$5.03/6.25 mL
NEAA	Gibco	$6.90/6.25 mL	$6.90/6.25 mL
IGF-1	Aladdin	0	$3.34/0.2 mL
bFGF	Aladdin	0	$2.41/0.05 mL
TGF-β	Aladdin	0	$3.34/0.005 mL
IL-6	GenScript	0	$3.95/0.05 mL
G-CSF	GenScript	0	$5.24/0.05 mL
**Total**		**$103.84/500 mL**	**$60.05/500 mL**

The price of each product was calculated based on the price purchased in China. As of 16 June 2025, 1 USD ≈7.18 CNY. PS, penicillin/streptomycin. Bold values represent the total cost of the culture media, specifically when using 20% Fetal Bovine Serum (FBS) or 5% FBS supplemented with PSFC.

### 3.7 Transcriptomic analysis confirms genomic stability and reveals molecular mechanisms underlying proliferation maintenance and enhanced transfection efficiency in 5% FBS-PSFC system

**FIGURE 8:**
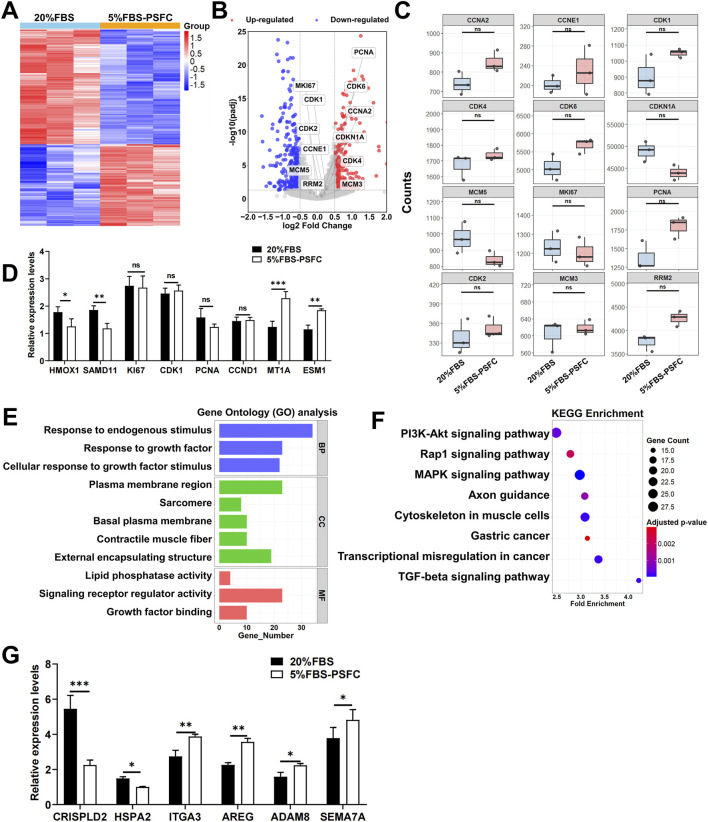
Transcriptomic profiling of PSCs cultured in 5% FBS-PSFC versus 20% FBS. **(A)** Heatmap of global gene expression patterns between 5% FBS-PSFC and 20% FBS conditions demonstrates high similarity between culture systems. Rows: genes; columns: biological replicates. Color scale represents Z-score normalized expression levels (red: high; blue: low), with clustering showing no systematic segregation by serum concentration. **(B)** Volcano plot analysis identifies 381 downregulated and 272 upregulated differentially expressed genes (DEGs). Gray points represent the majority (97.9%) of non-significant transcripts, confirming overall transcriptional stability. **(C)** Boxplots of normalized read counts for representative genes confirm consistent expression of proliferation markers between 5% FBS-PSFC and 20% FBS conditions. **(D)** The RT-qPCR validation of selected differentially expressed genes (DEGs) corroborates the findings from RNA sequencing, demonstrating a significant upregulation of *MT1A* and a downregulation of *SAMD11* in 5% FBS-PSFC, while the proliferation markers remain stable. **(E)** GO enrichment analysis reveals DEGs primarily associate with growth factor responsiveness (BP, blue), sarcomere organization (CC, green), and signaling receptor activity (MF, red). **(F)** KEGG pathway analysis highlights significant enrichment (FDR <0.05) in PI3K-Akt signaling (large dot, bright color) and moderate enrichment in MAPK/Rap1 pathways. **(G)** RT-qPCR analysis revealed downregulation of *CRISPLD2* and *HSPA2* alongside upregulation of *ADAM8* and *AREG*, consistent with ECM remodeling and improved plasmid accessibility. Concurrent elevation of *ITGA3* and *SEMA7A* suggests activation of endocytosis-related pathways. Data are shown as mean ± SD, n = 3. *P < 0.05, **P < 0.01, ***P < 0.001. ns indicates statistical non-significance.

To elucidate the molecular mechanisms underlying the maintenance of genomic stability, sustained proliferation, and enhanced transfection efficiency in the 5% FBS-PSFC system, we conducted a comprehensive RNA sequencing (RNA-seq) analysis of PSCs and C2C12 myoblasts. Heatmap visualization revealed comparable global gene expression patterns between the two serum conditions in both cell types (Figure 8A; Supplementary Figure S3A). Differential expression gene (DEG) analysis further indicated a remarkably low number of significantly altered genes, even under stringent thresholds: only 272 upregulated and 381 downregulated genes were identified in PSCs (Figure 8B), while merely 88 upregulated and 109 downregulated genes were detected in C2C12 cells (Supplementary Figure S3B), representing only 2.1% and 1.3% of the total detected transcripts, respectively. Notably, key proliferation-associated genes, including *MKI67*, *CCND1*, and *PCNA*, exhibited no substantial expression changes between conditions (Figure 8C; Supplementary Figure S3C). RT-qPCR validation of randomly selected DEGs (including upregulated, downregulated, and non-significance genes) demonstrated excellent concordance with RNA-seq data (Figure 8D; Supplementary Figure S3D), providing robust confirmation of the reliability of the RNA-seq results.

Gene Ontology (GO) enrichment analysis in PSCs identified a distinct cluster of growth-factor-responsive terms, including “Response to growth factor” and “Growth factor binding,” alongside “Plasma membrane region” (Figure 8E). This combination of annotations indicates that PSFC retains the capacity for extracellular cue recognition and maintains membrane integrity under 5% FBS-PSFC conditions, both of which are essential for efficient DNA uptake. In parallel, C2C12 myoblasts exhibited significant enrichment in cytoskeletal regulators, such as the “Integrin complex” and “Actin filament,” as well as cell-adhesion modules like the “Connexin complex” (Supplementary Figure S3E). This suggests an adaptive reorganization of the cortical actin network and focal adhesions, thereby facilitating endocytosis and subsequent plasmid internalization. KEGG pathway mapping corroborated these observations. In PSCs, the classic drivers of proliferation and survival, namely the PI3K-Akt, MAPK, and Rap1 signaling axes, were selectively activated (Figure 8F). In contrast, C2C12 myoblasts exhibited significant upregulation of the focal adhesion and regulation of actin cytoskeleton pathways, both of which govern membrane dynamics and clathrin-independent endocytosis (Supplementary Figure S3F). Based on KEGG pathway analysis, we focused on validating genes within the PI3K-Akt and focal adhesion pathways that exhibited significant expression changes. Notably, in PSCs, the transcript levels of *CRISPLD2* were downregulated, while those of *ADAM8* and *AREG* were upregulated (Figure 8G). This pattern has been previously associated with ECM remodeling and enhanced plasmid accessibility in other systems (Hao et al., 2020). Similarly, elevated expression levels of *ITGA3* and *SEMA7A* were correlated with membrane protein-mediated endocytosis pathways, although protein-level validation remains to be investigated (Figure 8G). Collectively, these transcriptional changes may collectively contribute to improved transfection efficiency under low-serum conditions.

## 4 Discussion

This study demonstrates that the PSFC formulation effectively sustains both proliferation and differentiation under low-serum conditions while significantly enhancing transfection efficiency. In contrast to other low-serum systems, such as Beefy-9 medium for bovine satellite cells (Stout et al., 2022), our method achieves comparable levels of proliferation with markedly reduced cytokine costs. Furthermore, the average gain of 16.9% in transfection efficiency exceeds the improvements typically achieved through lipid-based delivery optimization alone (Zuris et al., 2015), indicating a synergistic effect between serum reduction and PSFC. Collectively, these advantages position our system as a highly cost-effective platform for cellular agriculture.

RNA-seq analysis demonstrated that PSFC effectively compensated for serum reduction through selective activation of key proliferative pathways, including PI3K-Akt and MAPK/Rap1 signaling, while maintaining transcriptomic stability. Notably, the differential expression of these genes accounted for only 2.1% of total transcripts in PSCs and 1.3% in C2C12 myoblasts when compared to high-serum controls. This aligns with earlier studies demonstrating that multi-factor combinations of FGF-2, TGF-β3, and IGF-1 enhance mesenchymal stem cell proliferation through the upregulation of cell cycle markers (Li et al., 2023). Additionally, it has been shown that bFGF-driven ERK1/2 activation is essential for myoblast expansion (Liu et al., 2024). However, PSFC uniquely integrates the immunomodulatory cytokines IL-6 and G-CSF, which we have validated as potent enhancers of PSC proliferation. IL-6, an exercise-induced factor, directly activates satellite cells (Otsuka et al., 2025), while G-CSF exerts paracrine effects that foster a pro-regenerative niche (Shen et al., 2024; Yang et al., 2024). Therefore, the 5% FBS-PSFC system represents a tailored strategy for porcine muscle cells and fibroblasts, which are challenging models for low-serum culture. This multifactorial approach not only compensates for serum reduction but also provides more precise control over the cellular microenvironment, marking a significant advancement toward the development of defined culture systems for cell type specificity.

Beyond sustaining proliferation, the 5% FBS-PSFC system significantly improved transfection efficiency by an average of 16.9% across all tested cell types. This enhancement can be attributed to the reduction of serum protein interference with transfection complexes (Wei et al., 2023), as well as the PSFC-mediated upregulation of genes associated with membrane fluidity, such as *ITGA3* and *SEMA7A* (Hu et al., 2023; Sun et al., 2024), along with endocytosis-related regulators including *ADAM8* and *AREG* (Arora et al., 2025). KEGG pathway analysis further revealed the activation of focal adhesion and actin cytoskeleton pathways, which facilitate nucleic acid internalization. Notably, the cytokine cocktail orchestrates a precisely timed cell cycle synchronization effect; the bFGF-mediated release from TGF-β-induced G1 arrest, followed by IGF-1-driven S phase entry, generates a transient population of cells in the G1/S transition phase, where membrane fluidity and endocytic activity are optimally conducive to transfection (Hefka Blahnova et al., 2020). Although these findings provide compelling evidence for the system’s effectiveness, further research is necessary to elucidate the specific mechanisms of action of the PSFC components. In brief, these findings position the 5% FBS-PSFC platform as a versatile tool for CRISPR-mediated genome editing (Kulkarni et al., 2024), the more efficient generation of stable cell lines (Chen et al., 2018), and enhanced production titers for viral vectors (Greene et al., 2021).

The 5% FBS-PSFC system presents preserving robust cell performance by reducing serum requirements by 50%–75%, thereby lowering medium costs than commercial serum-free alternatives (Table 4). Additionally, when evaluated using a 3D scaffold of GelMA hydrogels, the PSCs exhibited robust proliferation rates and maintained their cytoskeletal organization, confirming the system’s capability to support complex 3D tissue growth. In comparison to existing 3D culture systems, such as those utilizing high serum concentrations (10%–20% FBS) for the expansion of porcine satellite cells (Zhu et al., 2022) and serum-free differentiation methods employed in bovine models (Messmer et al., 2022), our 5% FBS-PSFC platform strikes an optimal balance between low cost, minimal serum dependence, and the preservation of myogenic capacity. This advantage highlights its significant potential for scalable cultured meat production. Notably, these economic and practical advantages are achieved without compromising cell proliferation functionality.

Despite these successes, our experiments demonstrated that reducing serum concentration to 2% FBS significantly impaired cell proliferation, underscoring the irreplaceable role of serum components in cell culture. Specifically, extracellular matrix (ECM) proteins and lipoproteins are critical for cell adhesion, signaling, and metabolic homeostasis (Karnieli et al., 2017; Sthijns et al., 2021). Although synthetic alternatives, such as RGD peptides, can partially mimic ECM functions, they do not possess the complexity of natural serum (Vallier, 2011). Future research should focus on the systematic identification of essential serum components through metabolomics and high-throughput screening, as well as the development of advanced delivery systems to better mimic physiological niches. Therefore, this study maintains a 5% FBS concentration, representing a pragmatic balance between cost reduction and functionality. It serves as a transitional solution until fully defined serum-free substitutes are developed (Somasekhar and Griffiths, 2023).

## 5 Conclusion

In this study, we established a novel proliferation synergy factor cocktail (PSFC) consisting of IGF-1, bFGF, TGF-β, IL-6, and G-CSF, which facilitates efficient cell culture under low-serum (5% FBS) conditions. This system effectively addresses significant limitations associated with traditional high-serum culture methods, such as undefined components, elevated costs, and increased contamination risks, while preserving essential cellular functions. The 5% FBS-PSFC system demonstrated broad applicability by sustaining robust proliferation of PSCs, PKFs, C2C12 myoblasts, and SSCs, while also maintaining myogenic differentiation capacity, as evidenced by normal myotube formation and the expression of MyoD and MyHC. Notably, PSFC supplementation increased transfection efficiency by 16.9% across all tested cell types and reduced serum requirements by 50%–75%, thereby lowering medium costs. Furthermore, the system demonstrated compatibility with 3D culture in GelMA hydrogels, underscoring its potential utility in cultured meat and regenerative medicine applications. Further RNA-seq analysis revealed that the there was no significant changes in the expression of cell proliferation-related genes which may be crucial for maintaining cell proliferation of this system. The enhancement of transfection efficiency is facilitated by the upregulation of genes related to membrane fluidity and factors that regulate endocytosis. In summary, the 5% FBS-PSFC platform achieves an optimal balance between biological performance and cost-effectiveness, providing a versatile solution for cell expansion, genetic manipulation, and large-scale biomanufacturing. This platform paves the way for standardized, low-serum culture systems in cellular agriculture, biomedical research, and therapeutic development.

## Data Availability

The datasets presented in this study can be found in online repositories. The names of the repository/repositories and accession number(s) can be found in the article/Supplementary Material.
