# Supplementary material for: A novel proliferation synergy factor cocktail maintains proliferation and improves transfection efficiency in muscle cells and fibroblasts under low-serum conditions
Source: Front Cell Dev Biol. 2025 Nov 17;13:1680263. doi: 10.3389/fcell.2025.1680263 (PMC12665707; doi:10.3389/fcell.2025.1680263)
Supplement: Supplementary file 1 [file DataSheet1.zip › Supplementary figures.docx]

**Supplementary figures**


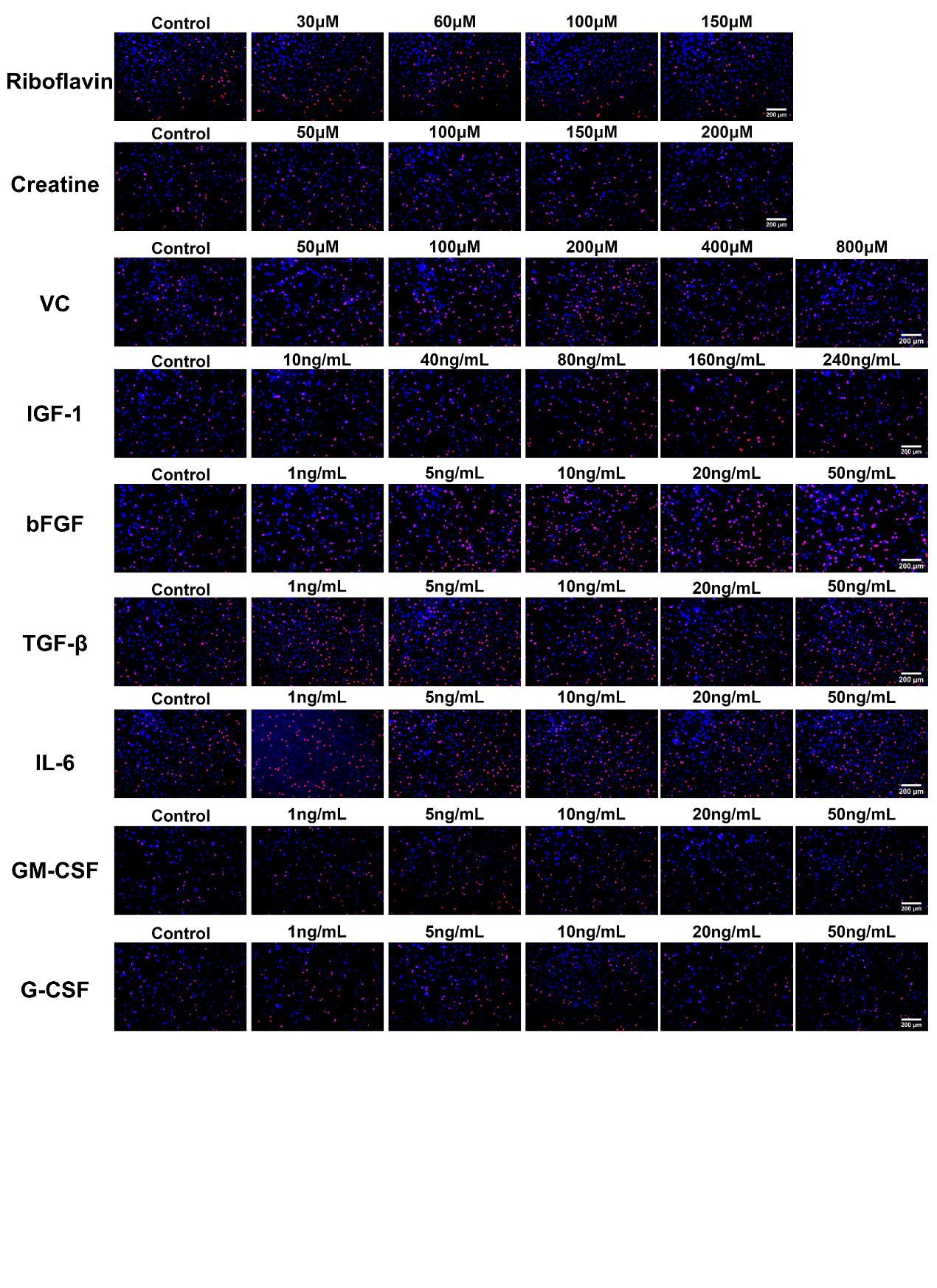


**Figure S1. EdU proliferation assay after 24-hour treatment with small molecules and cytokines.** Neither riboflavin nor creatine treatment showed significant mitogenic effects compared to controls. Dose-dependent proliferation enhancement was observed for VC (optimal concentration: 100 μM), IGF-1 (40 ng/mL), bFGF (10 ng/mL), TGF-β (5 ng/mL), IL-6 (10 ng/mL), GM-CSF (10 ng/mL), and G-CSF (10 ng/mL). Data are shown as mean ± SD, n = 3.

**
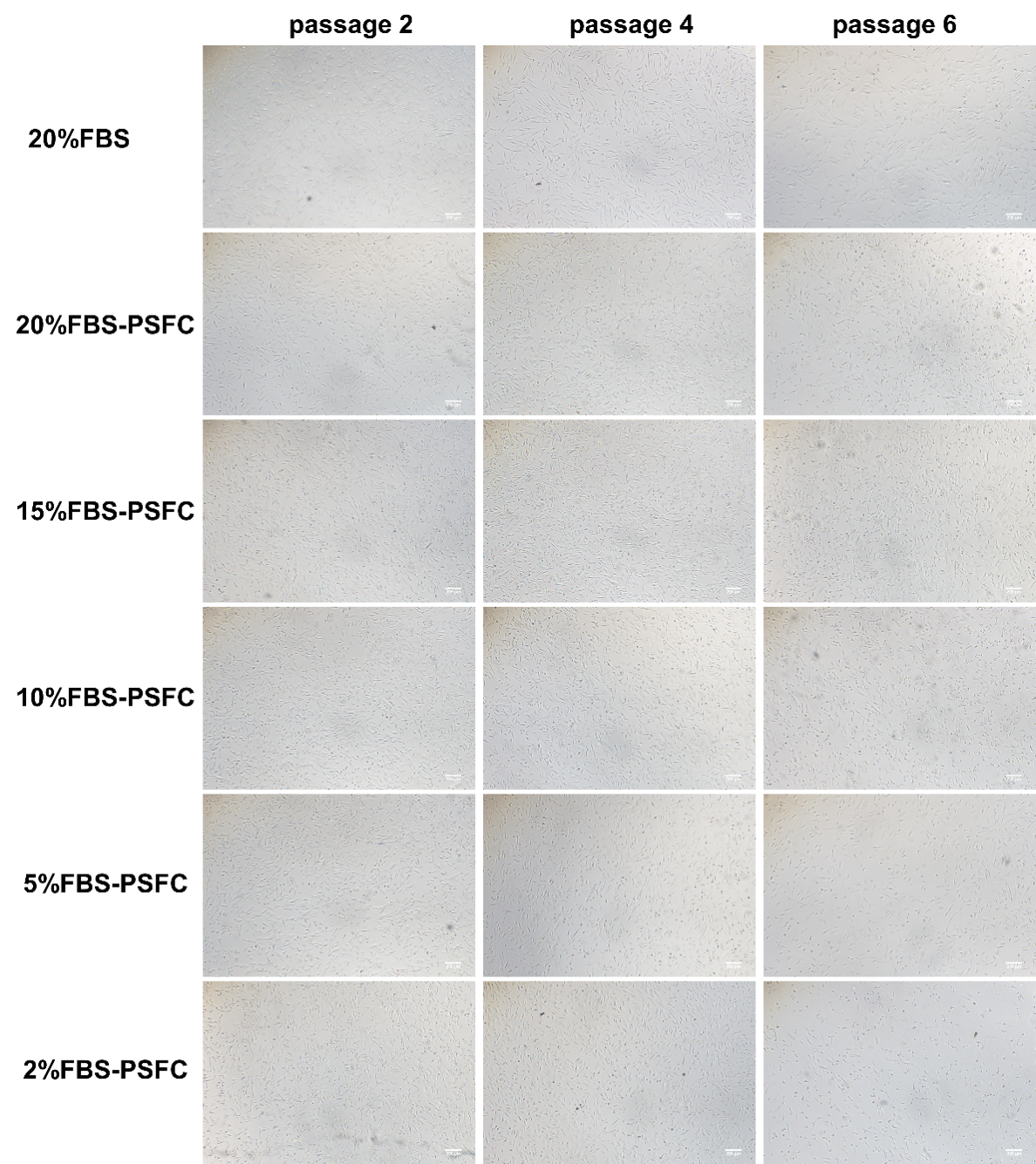
**

**Figure S2** **Bright-field microscopy was employed for morphological assessment across different passages (P2, P4, P6).** **n = 3.**


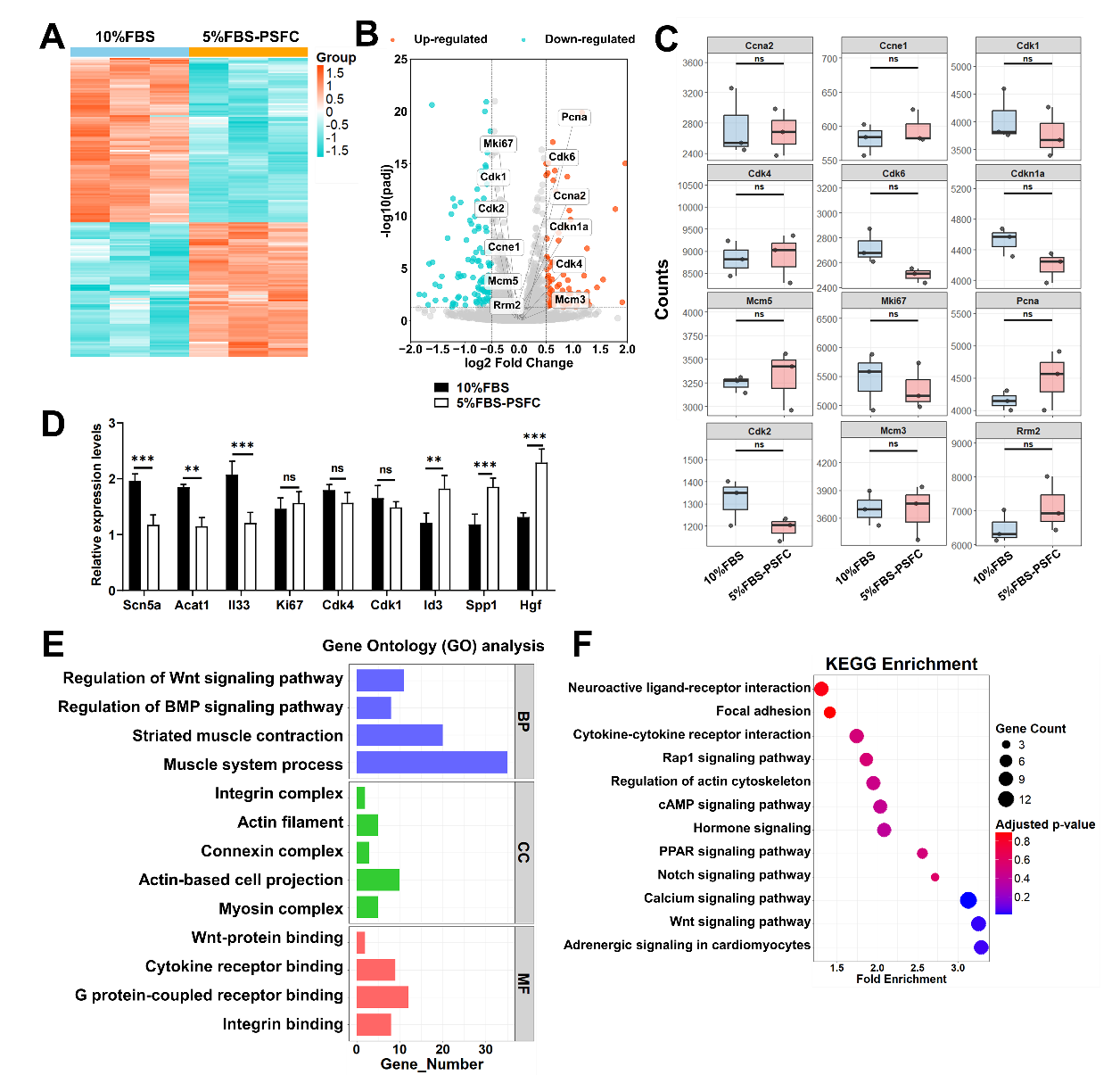


**Figure S3. Transcriptomic analysis of C2C12 cells cultured in 5% FBS-PSFC versus 10% FBS.** (A)Heatmap of global gene expression patterns between 5% FBS-PSFC and 10% FBS conditions demonstrates high similarity between culture systems. Rows: genes; columns: biological replicates. Color scale represents Z-score normalized expression levels (orange: high; green: low). (B) Volcano plot identifies 109 downregulated and 88 upregulated DEGs. Gray points represent 98.7% of transcripts showing non-significant changes, paralleling porcine data. (C) Boxplots of normalized read counts for representative genes confirm consistent expression of proliferation markers between 5% FBS-PSFC and 20% FBS conditions. (D) The RT-qPCR validation verifies RNA-seq predictions: *Scn5a* shows significant downregulation while *Id3* exhibits upregulation in 5% FBS-PSFC, with cell cycle markers maintaining consistent expression. (E) GO enrichment demonstrates DEG associations with muscle contraction (BP, blue), integrin complexes (CC, green), and cytokine binding (MF, red), differing from porcine functional profiles. (F) KEGG analysis detects significant enrichment in calcium signaling (FDR < 0.05) and marginal Wnt pathway involvement. Data are shown as mean ± SD, n = 3. **P*< 0.05, ** *P* < 0.01, *** *P* < 0.001. ns indicates statistical non-significance.
